# Trichotillomania in a dementia case

**DOI:** 10.1590/S1980-57642011DN05010011

**Published:** 2011

**Authors:** Leonardo Caixeta, Danielly Bandeira Lopes

**Affiliations:** 1 M.D, Ph.D. Associate Professor of Neuroscience, Federal University of Goiás (UFG), Goiânia GO, Brazil. Coordinator, Cognitive and Behavioral Neurology Unit, Hospital das Clínicas (UFG).; 2 MSc, Behavioral and Cognitive Neurology Unit, Hospital das Clínicas, Federal University of Goiás, Goiânia GO, Brazil.

**Keywords:** Trichotillomania, impulse-control disorder, dementia, Binswanger’s disease, perseveration

## Abstract

We report an 87-year-old male case of hair pulling associated with a white-matter vascular dementia (Binswanger’s disease). Trichotillomania in our case did not resolve using mirtazapine or anticholinesterasic medication. Trichotillomania seems to be related to a form of perseveration associated with dementia. The findings in this case suggest the abnormality involving white matter in the pathogenesis of trichotillomania, may constitute a defect in connectivity in the right frontal-subcortical circuit.

## Abstract (pt)

Relatamos o caso de um homem de 87 anos com tricotilomania associada com uma demência vascular da substância branca (doença de Binswanger). Tricotilomania no nosso caso não se resolveu com mirtazapina nem com medicação anticolinesterásica. Tricotilomania parece se relacionar a uma forma de perseveração associada à demência. Podemos sugerir com este caso que a alteração que envolve a substância branca na patogênese da tricotilomania pode ser uma falha na conectividade no circuito fronto-subcortical direito.

Although several studies have revealed the role of impulsivity in mental disorders, few studies have investigated its variants in dementia.^1^ Impulsivity is defined as the failure to resist a drive or stimulus, or in a personality dimension as the inability to resist the desire to harm one’s self or others.^2^ Impulsivity can be a psychopathological structural part of many mental disorders and, although not defined in detail in DSM-IV, it is mentioned as a diagnostic criterion in several mental disorders such as impulse control disorders (pathological gambling, intermittent explosive disorder, pyromania, kleptomania and trichotillomania), impulsive aggressive disorders of personality (borderline, antisocial, histrionic and narcissistic), manic episodes of bipolar disorder, attention deficit hyperactivity disorder (ADHD), neurological disorders with behavioural disinhibition and substance abuse.^2^

Trichotillomania is a disorder characterized by repetitive hair pulling, leading to noticeable hair loss and functional impairment.^3^ Its neurobiological basis is not fully understood.^4^ Whole-brain trichotillomania neuroimaging studies are lacking.

We report a case of hair pulling associated with a white-matter vascular dementia: Binswanger’s disease.

## Case report

An 87-year-old right-handed Afro-Brazilian male with four years of schooling, was first referred to the dementia outpatient division of the Hospital das Clínicas of the Federal University of Goiás in December 2006. He presented with progressive cognitive deterioration, mainly memory impairment and executive dysfunction (difficulties in planning, sequencing, abstraction and goal-directed behavior), as well as a history of personality changes and depressive traits that began in 2002. At this time, he had a CDR of 1 and no trichotillomania.

At home, his sister (and main caregiver) reported progressive loss of autonomy, neglect of hygiene, abandonment of personal interests, intense apathy (he lay in bed all day long, locked in his room).

The patient was diagnosed with Binswanger’s disease, a type of vascular dementia and depression, probably of vascular origin. Rivastigmine (progressively increased to 12 mg daily) and mirtazapine (45 mg daily) were prescribed, with moderate benefit in memory and resolution of depressive symptoms, respectively.

On November 2008, now with CDR 2, he began a repetitive behavior characterized by hair pulling of his beard, all day, every day, persisting despite family counseling. The patient gave no explanation for this act, and denied any feeling of tension prior to the act or deriving any pleasure from the act. Additionally, he reported no pain, and had no insight regarding its compulsive nature or potential harmful consequences to his skin. There was no evidence of any delusional beliefs related to his hair-pulling behaviors. No other psychotic symptoms were elicited. There was no apparent precipitating event prior to these behaviors, no history of impulsive behaviors or other obsessive-compulsive behaviors were elicited. No history of OCD was disclosed. Trichotillomania persists until the present day.

At last visit, he was self- and allo-psychically disoriented, presenting apathy, severe amnesia and continued trichotillomania, even when requested to stop.

Important antecedents included diabetes and high blood pressure, both well controlled. The only relevant familial antecedent was an uncle who had “Alzheimer’s disease” when he was 89 years old, according to information from his sister. There were no relevant familial psychiatric antecedents.

He presented almost complete blindness (only 30% of visual acuity in his right eye). On neurological examination, he presented with fluent speech, brisk reflexes and Babinski’s sign on the left side, exalted primitive reflexes, bilateral paratonia, with a hesitant gait (because of his blindness) but with no Romberg sign or deficits in cranial nerves, coordination, motor, and sensory systems. His psychopathological examination revealed significant apathy, reduced verbal output, lack of insight of his compulsive behavior, no depressive mood or anxiety and with preservation of social rules and adequacy. His MMSE score was 10 points (certainly impacted by his blindness) and he scored 2 on the CDR and 25 on activities of daily living (Pfeffer et al.^12^). The patient’s neuropsychological exam showed marked memory deficits (but with regular performance on recognition), severe executive dysfunction (perseveration and reduced mental control, abstraction, conceptualization, planning, initiation, cognitive flexibility, conceptualization and set shifting), moderate attention deficits, and mild ideomotor apraxia. The visuoconstructional and visuospatial tests were hindered by his blindness. He recognized objects placed in his hands. No language deficits were notable, except for reduced verbal fluency.

**Figure 1:**
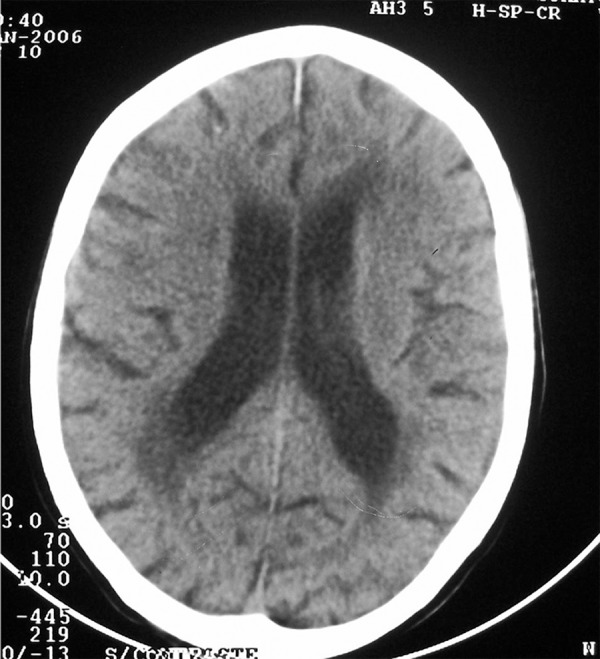
CT showing leukoaraiosis (periventricular hypodensity) suggestive of Binswanger’s disease.

Brain CT showed leukoaraiosis evidenced by periventricular hypodensity, accompanied by ventricular enlargement, suggestive of a subcortical pathology (Figure 1). Metabolic workup for treatable causes of dementia revealed no abnormalities that could contribute to his cognitive deficits or mood symptoms.

## Discussion

Trichotillomania is a poorly understood complex disorder of multifaceted pathology which often requires an interdisciplinary approach for management.^4^ This psychopathogical phenomenon seems to be rare in dementia patients. Trichotillomania was previously reported in the literature only by Mittal et al.^5^ who presented a case of trichotillomania associated with frontal dementia. In both present and previously reported cases, the symptom appeared with dementia progression. While having some similarities with obsessive-compulsive disorder, compelling differences between these have also been noted,^3,4^ and our patient had no other obsessive-compulsive symptoms.

According to DSM-IV,^6^ diagnostic criteria of trichotillomania include:

[a] recurrent pulling out of one’s hair resulting in noticeable hair loss;
[b] an increasing sense of tension immediately before pulling out hair or when attempting to resist the behavior;
[c] pleasure, gratification, or relief when pulling out hair;
[d] the disturbance is not better accounted for by another mental disorder and is not due to a general medical condition (e.g., a dermatological condition);
[e] the disturbance causes clinically significant distress or impairment in social, occupational, or other important areas of functioning. Our patient fulfilled all these criteria, except for relief of tension when pulling out his hair, which proves hard to verify because of the difficulty in accessing the inner world of demented patients. This aspect may lack in the full clinical picture in dementia patients.

In fact, many patients with trichotillomania do not manifest the full DSM-IV criteria or do so intermittently.^4^ Another aspect not generally observed in trichotillomania patients, but present in our dementia case, was loss of insight of the altered behavior, thus indicating frontal dysfunction.

The most significant neurobiological finding in our case was white matter pathology. Using magnetic resonance imaging (MRI), Mittal et al.^5^ also revealed ischemic lesions in the right cerebral hemisphere in deep white matter of their dementia case. There are no other neurobiological data available in the literature concerning trichotillomania in dementia. O’Sullivan et al.^7^ reported reduced left putamen volumes in a sample of 10 patients with trichotillomania vs. 10 healthy controls. However, these results were not replicated by other authors^8^ who found no evidence for caudate volume abnormalities in 7 patients with trichotillomania compared with 12 controls using magnetic resonance imaging (MRI). Keuthen et al.^9^ reported reduced cerebellar volumes in a sample of 14 patients with trichotillomania vs. 12 controls, using MRI but their results were also not replicated. Chamberlain et al.,^10^ in a study investigating patients with trichotillomania, showed increased grey matter densities in the left striatum, left amygdalohippocampal formation, and in multiple cortical regions bilaterally (including cingulate, supplementary motor, and frontal), concluding that trichotillomania was associated with structural grey matter changes in neural circuitry implicated in habit learning, cognition and affect regulation. In our case, we propose the hypothesis that trichotillomania, a form of impulsive-control disorder, is related to a kind of perseveration (also evident on neuropsychological tests) that in turn is connected with dysfunctional frontosubcortical circuits located in the non-dominant hemisphere. In one study,^11^ trichotillomania patients evaluated neuropsychologically showed increased perseveration on the Object Alternation Task, suggesting difficulties with response flexibility. Further studies are necessary to confirm an association between trichotillomania and perseveration, particularly in dementia sufferers.

The trichotillomania in our case cannot be attributed to depressive self harm, since the patient had previously been treated for depression when this symptom appeared and was euthymic at onset. Our results also suggest that trichotillomania in dementia sufferers does not resolve when treated with mirtazapine or anticholinesterasic medication, since these drugs did not prevent the onset of trichotillomania in our patient.

The results of this case suggest that the abnormality involving white matter in the pathogenesis of trichotillomania may constitute a defect in connectivity in the frontal-subcortical circuit.
